# A Meta-Analysis for Association of XRCC3 rs861539, MTHFR rs1801133, IL-6 rs1800795, IL-12B rs3212227, TNF-α rs1800629, and TLR9 rs352140 Polymorphisms with Susceptibility to Cervical Carcinoma

**DOI:** 10.31557/APJCP.2021.22.11.3419

**Published:** 2021-11

**Authors:** Seyedeh Fatemeh Parsaeian, Fatemeh Asadian, Mojgan Karimi-Zarchi, Sepideh Setayesh, Atiyeh Javaheri, Razieh Sadat Tabatabaie, Seyed Alireza Dastgheib, Hossein Golestanpour, Hossein Neamatzadeh

**Affiliations:** 1 *Department of Obstetrics and Gynecology, Shahid Sadoughi University of Medical Sciences, Yazd, Iran. *; 2 *Department of Medical Laboratory Sciences, School of Paramedical Science, Shiraz University of Medical Sciences, Shiraz, Iran. *; 3 *Endometriosis Research Center, Iran University of Medical Sciences, Tehran, Iran. *; 4 *Department of Obstetrics and Gynecology, Firoozgar Hospital, Iran University of Medical Sciences, Tehran, Iran. *; 5 *School of Medicine, Shiraz University of Medical Sciences, Shiraz, Iran. *; 6 *Department of Medical Genetics, School of Medicine, Shiraz University of Medical Sciences, Shiraz, Iran.*; 7 *Department of Genetics, Marvdasht Branch, Azad University, Marvdasht, Iran. *; 8 *Biotechnology Research Center, International Campus, Shahid Sadoughi University of Medical Sciences, Yazd, Iran. *; 9 *Mother and Newborn Health Research Center, Shahid Sadoughi University of Medical Sciences, Yazd, Iran. *; 10 *Department of Medical Genetics, Shahid Sadoughi University of Medical Sciences, Yazd, Iran. *

**Keywords:** Cervical carcinoma, cervical cancer, meta-analysis, gene, polymorphism

## Abstract

**Background:**

In spite of substantial declines in both incidence and mortality rates in the past 50 years, cervical cancer remains one of the leading causes of cancer associated mortality among women globally. We performed this meta-analysis to explore the role of XRCC3 rs861539, MTHFR rs1801133, IL-6 rs1800795, IL-12B rs3212227, TNF-α rs1800629 and TLR9 rs352140 polymorphism with susceptibility to cervical carcinoma.

**Methods:**

The search databases include PubMed, SciELO, MedRxiv, Web of Science, Scopus, Cochrane Library, China National Knowledge Infrastructure, and China Biology Medicine disc up to 30 June 2021. The language is limited to English and Chinese. The comparison between the polymorphisms and cervical cancer was assessed using pooled odds ratio (OR) and 95% confidence interval (CI). The data are statistically analyzed by Comprehensive Meta-Analysis (CMA) 2.0 software.

**Results:**

A total of 59 studies including seven studies with 1,112 cases and 1,233 controls on XRCC3 rs861539, 14 studies with 2,694 cases and 3349 controls MTHFR rs1801133, four studies with 1,121 cases and 1,109 controls on IL-12B rs3212227, seven studies with 1,452 cases and 2,186 controls on IL-6 rs1800795, 20 studies with 4,781 cases and 4909 controls on TNF-α rs1800629, and seven studies with 1743 cases and 2292 controls on TLR9 rs352140 were included. There was a significant association between XRCC3 RS861539, TNF-α rs1800629, and IL-6 rs1800795 polymorphisms and an increased risk of cervical carcinoma in overall population. However, the MTHFR rs1801133, IL-12B rs3212227 and TLR9 rs352140 polymorphisms were not associated.

**Conclusion:**

The pooled analysis showed that XRCC3 RS861539, TNF-α rs1800629, and IL-6 rs1800795 were associated with cervical carcinoma susceptibility, but not MTHFR rs1801133, IL-12B rs3212227 and TLR9 rs352140 polymorphisms.

## Introduction

Cancer is one of the main public health problems globally with about 18.1 million new cancer cases and 9.6 million cancer deaths in 2018 (Ghelmani et al., 2021; Jarahzadeh et al., 2021; Antikchi et al., 2021). The World Health Organization (WHO) states cervical cancer is the second most frequent cancer among women worldwide (Hamadani et al., 2017; Da Silva et al., 2021) with an estimated 570,000 new cases and 311,000 deaths in 2018 worldwide (Yi et al., 2020). It is reported that 85% of cervical cancer cases and 87% of the cervical cancer deaths occur in less developed countries (WHO/ICO, 2010). With over 500,000 cases of cervical cancer reported each year, nearly 80 percent of those are in developing countries, including Africa with 68,000, an estimated 77,000 in Latin America and the Caribbean, and 245,000 in Asia (Arbyn et al., 2020). Cervical cancer is driven by persistent infection with one of 15 carcinogenic human papillomavirus (HPV) types (Ghaemmaghamiet al., 2008; Chen et al., 2011). However, the impact of HPV type and intratypic variants on patient outcomes is far less understood (Karimi-Zarchi et al., 2013; Baghestani et al., 2018; Rader et al., 2019).

To date, the occurrence and development of cervical cancer is suggested to be associated with persistent HPV infection (Ghaemmaghami et al., 2008; Zarchi et al., 2010; Pal and Kundu, 2020). The DNA of HPV integrates into the host cell genome and disrupts the open reading frame and causes overexpression of E6 and E7 genes (Binesh et al., 2012; Soheili et al., 2016). However, the specific molecular mechanisms and potential single gene require further studies. Familial based studies and evaluation of inherited genetic variations revealed that host genetic factors have a role in cervical cancer pathogenesis (Yang et al., 2020; Ahmadi et al., 2021). Recently, a comprehensive review study showed that CDK1, CCNB1, ITGB1, FN1, MMP9 and STAT1 played different roles in the progression of cervical cancer through different signaling pathways (Sayad, Ahmadi, Nekouian, et al., 2020; Yi et al., 2020). Moreover, studies have shown that cervical cancer has a heritable genetic component. However, as other complex disease, the identified loci only explain a minority of the risk of cervical cancer. This gap of the heritability explained by genetic markers genome-wide association studies and the heritability identified in familial studies has been termed as ‘missing heritability’ (Chen et al., 2016; Leo et al., 2017; Sayad, Ahmadi, Moradi, et al., 2020). Thus, our understanding of the genetic basis of cervical cancer is still limited. In this meta-analysis, we explore the role of XRCC3 rs861539, MTHFR rs1801133, IL-6 rs1800795, IL-12B rs3212227, TNF-α rs1800629, and TLR9 rs352140 polymorphisms in susceptibility to cervical cancer.

## Materials and Methods

*Literate Search Strategy*

We performed a comprehensive literature search on PubMed, Scopus, China National Knowledge Infrastructure, Wanfang, VIP Information Chinese Journal Service Platform, and China Biology Medicine disc, PubMed, EMBASE, Web of Science and the Cochrane Library, Google Scholar, Cochrane Library, EMBASE, Scientific Information Database (SID), WanFang, VIP, Chinese Biomedical Database (CBD), Scientific Electronic Library Online (SciELO), China National Knowledge Infrastructure (CNKI), IranDoc and Egyptian Knowledge Bank (EKB) Journals database to identify all relevant studies on the association of XRCC3 rs861539, MTHFR rs1801133, IL-6 rs1800795, IL-12B rs3212227, TNF-α rs1800629 and TLR9 rs352140 with cervical cancer up to 30th June 2021. The following terms were used in various combinations in this search: (‘’Uterine Cervical Neoplasm‘’ OR ‘’Cervix Cancer’’ OR ‘’Cervical Cancer’’ OR ‘’Cervical Neoplasm’’ OR ‘’Cervical Carcinoma’’ OR ‘’Squamous Cell Carcinoma’’ OR ‘’Adenocarcinoma’’) AND (‘’X-Ray Repair Cross Complementing 3’’ OR ‘’XRCC3’’ OR ‘’rs861539’’ OR ‘’g.4880C>G’’ OR ‘’c.-237C>G’’ OR ‘’n.54-321G>C’’) AND (‘’Methylene tetrahydrofolate reductase’’ OR ‘’MTHFR’’ OR ‘’677C>T’’ OR ‘’rs1801133’’ OR ‘’g.14783C>T’’ OR ‘’c.788C>T’’ OR ‘’p.Ala263Val’’ OR ‘’p.Ala222Val’’) AND (‘’Interleukin 12’’ OR ‘’IL-12’’ OR ‘’1188A>C’’ OR ‘’rs3212227’’ OR ‘’ g.158742950T>G’’ OR ‘’ g.19532A>C’’ OR ‘’ c.*159A>C’’) AND (‘Interleukin 6’’ OR ‘’IL-6’’ OR ‘’-174G>C’’ OR ‘’rs1800795’’ OR ‘’ g.4880C>G’’ OR ‘’c.-274C>G’’ OR ‘’ n.54-321G>C’’) AND (‘’Toll like receptors’’ OR ‘’TLR’’ ‘’ OR ‘’rs352140’’ OR ‘’2848G/A’’) AND (‘’Gene’’ OR ‘’Single-Nucleotide Polymorphism” or “SNP” OR ‘’Polymorphism’’ OR ‘’Genotype’ OR ‘’Allele’’ OR ‘’Variant’’ OR ‘’Variation’’ OR ‘’Mutation’’ OR ‘’Mutant’’). The search was carried out in English, Chinese and Persian. When overlapping data on the same cases were included in more than one publication, only the one with the larger sample size was selected. Moreover, the reference list of the retrieved studies and reviews manually checked to identify more potential eligible studies.

*Eligibility criteria*

To obtain the papers, the following criteria should be met to include the papers in our study: 1) studies with case-control or cohort design; 2) studies reported original data; 3) studies appraised association of XRCC3 rs861539, MTHFR rs1801133, IL-6 rs1800795, IL-12B rs3212227, TNF-α rs1800629 and TLR9 rs352140 polymorphisms with cervical cancer; and 4) studies with available and sufficient data for calculating an odds ratio (OR) with 95% confidence interval (CI). The following exclusion criteria were also used: 1) studies on other polymorphism at XRCC3, MTHFR, IL-12B, IL-6, and TNF-α genes; 2) animal studies or in vitro studies; 3) studies with in sufficient data on genotype frequencies or which the number of genotypes and alleles could not be ascertained; 4) linkage studies; 5) family based studies including sibling, twins and trios-parents studies; 6) abstracts, case reports, commentaries, editorials, conference articles, reviews, proceedings and meta-analyses; and 7) duplicates studies or overlapping data.

*Data Extraction*

Two authors reviewed and extracted necessary information independently in accordance with our inclusion criteria. For conflicting data, the authors carried out discussions until a consensus was reached. If they could not reach a consensus, disagreement was resolved by the third author who participated in the discussion. For each eligible study the following data was collected: first author name, year of publication, country of origin, ethnicity (Caucasian, Asian, African, Mixed populations), genotyping methods, sample size, allele and genotype frequency of XRCC3 rs861539, MTHFR rs1801133, IL-12B rs3212227, IL-6 rs1800795, TNF-α rs1800629 and TLR9 rs352140 polymorphisms in cervical cancer cases and controls, Minor Allele Frequency (MAFs) and Hardy-Weinberg equilibrium (HWE) in healthy controls. In this meta-analysis different case-control groups or cohorts in one publication were considered as independent studies. We did not define any minimum sample size to include in this meta-analysis. The ‘‘mixed’’ group means mixed or unknown populations. If more than one study was published by the same author(s) using repeated or overlapped data, the studies with the largest sample size or the most recently published study was included to the meta-analysis. If selected articles did not reported necessary data the corresponding authors was contacted by email to request the missing data.

*Statistical Analysis*

The comparison between the XRCC3 rs861539, MTHFR rs1801133, IL-6 rs1800795, IL-12B rs3212227, TNF-α rs1800629 and TLR9 rs352140 polymorphism and cervical cancer was assessed using pooled odds ratio (OR) and 95% confidence interval (CI). The pooled data were calculated under five genetic models, i.e., allele (B vs. A), homozygote (BB vs. AA), heterozygote (BA vs. BB), dominant (BB+BA vs. AA) and recessive (BB vs. BA+AA), in which a ‘‘A’’ denotes a major allele; ‘‘B’’ denotes a minor allele. The *χ*^2^ test and I^2^ statistics were used to assess whether there was between-study heterogeneity, in which P >.10 and I^2^ < 50% could be considered that there was no statistical heterogeneity between the research results. The fixed effect model was selected for data consolidation; P ≤.10 and I^2^ ≥50% could be considered that there was statistical heterogeneity between the research results, and a random effect model was used for data consolidation. If there was significant heterogeneity among studies, the random effects model (DerSimonian and Laird) was used; otherwise, the fixed-effects model (Mantel and Haenszel) was acceptable. The Hardy-Weinberg equilibrium of the control group was evaluated using the *χ*^2^ test, and the expected and actual genotype frequencies of the control group were compared. In this meta-analysis, P-values of ≤.05 were considered statistically significant (Azadi-Yazdi et al., 2017; Mirjalili et al., 2019; Dastgheib et al., 2020). In addition, the sensitivity analysis was performed by excluding HWE-violating studies. Potential publication bias was evaluated using the Egger’s linear regression test and Begg’s quantitative test. The asymmetric plot of Egger’s test and the P-value of Begg’s test less than 0.05 were considered a significant publication bias. If there was evidence of publication bias (P<0.05), trim and fill method was applied to adjust for the effect of publication bias (Jafari et al., 2020). All statistical analyses were performed using Comprehensive Meta-Analysis (CMA) Software version 2.0 (Biostat, Englewood, USA). All tests were two-sided, and the P< 0.05 was considered statistically significant.

## Results

*Characteristics of the Enrolled Studies*

The flowchart of selection of studies and reasons for exclusion is presented in Figure 1. There were 1,013 articles relevant to our search words and 395 duplicated studies were excluded. Then, 207 studies removed after reading titles and abstracts. Following the inclusion exclusion criteria 352 studies were excluded. Finally, a total of 59 studies were included in this meta-analysis. There are seven studies with 1112 cases and 1233 controls on XRCC3 rs861539 (He et al., 2008; Xiao, 2009; Settheetham-Ishida et al., 2011; Djansugurova et al., 2013; Pérez et al., 2013; Al-Harbi et al., 2017), 14 studies with 2694 cases and 3349 controls MTHFR rs1801133 (Lambropoulos et al., 2003; Sull et al., 2004; Kang et al., 2005; Zoodsma et al., 2005; Delgado-Enciso et al., 2006; Ma et al., 2006; Nandan et al., 2008; Shekari et al., 2008; Kohaar et al., 2010; Yang et al., 2011; Prasad and Wilkhoo, 2011; Tong et al., 2011; Mostowska et al., 2011; von Keyserling et al., 2011), four studies with 1121 cases and 1109 controls on IL-12B rs3212227 (Chen et al., 2009; Do Carmo Vasconcelos De Carvalho et al., 2012; Roszak, Mostowska, et al., 2012), seven studies with 1452 cases and 2186 controls on IL-6 rs1800795 (Nogueira De Souza et al., 2006; Gangwar et al., 2009; Grimm et al., 2011; Shi et al., 2013; de Lima Júnior et al., 2016; Pu et al., 2016; Zidi et al., 2017), 20 studies with 4,781 cases and 4909 controls on TNF-α rs1800629 (Stanczuk et al., 2019; Jang et al., 2001; Calhoun et al., 2002; Gostout et al., 2003; Deshpande et al., 2005; Duarte et al., 2005; Govan et al., 2006; Kohaar et al., 2007; Singh et al., 2009; Zu et al., 2010; Ivansson et al., 2010; Zuo et al., 2011; Wang et al., 2011, 2012; Badano et al., 2012; Barbisan et al., 2012; Sousa et al., 2014; Roszak et al., 2015; Zidi et al., 2015) and seven studies with 1743 cases and 2292 controls on TLR9 rs352140 (Pandey et al., 2011; Roszak, Lianeri, et al., 2012; Lai et al., 2013; Bi, 2014; Zidi et al., 2016; Jin et al., 2017). The details of included studies were shown in Tables 1 and 2. All those 22 studies were reported in English and Chinese. Among those 59 studies, 31 studies were from Asian populations, 18 studies from Caucasian populations, five studies from African populations, and five studies were from mixed populations. The studies have been carried out in China, Thailand, Argentina, Kazakhstan, Brazil, Saudi Arabia, Greece, Korea, Netherlands, Mexico, India, Poland, Germany, Austria, Tunisia, Zimbabwe, USA, South Africa, Sweden, and Portugal. The sample size of cases ranged from 21 to 636, while the sample size of controls ranged from 73 to 800 in the controls. Eight different methods including AS-PCR, PCR-RFLP, Direct Sequencing, SnapShot, TaqMan, LDR-PCR, ARMS-PCR, qRT-PCR and HMR were used to genotyping those polymorphisms. Hardy-Weinberg equilibrium (HWE) test was calculated for all publications and P<0.05 was considered as a departure from HWE (Table 1).

*Quantitative Data Synthesis*

*XRCC3 RS861539*

The summary for the association of XRCC3 RS861539 polymorphism with cervical cancer risk are shown in Table 3. Pooled data revealed that XRCC3 RS861539 polymorphism was significantly associated with susceptibility to cervical cancer under the heterozygote genetic model (TC vs. CC: OR = 1.00, 95% CI 1.066-1.585, p = 0.009). Moreover, stratified analysis by ethnicity revealed that the polymorphisms was significantly associated with cervical cancer in Asian women under three genetic models, i.e., allele (T vs. C: OR= 1.302, 95% CI 1.076-1.576, P= 0.007), heterozygote (TC vs. CC: OR = 1.441, 95% CI 1.113-1.867, p = 0.006) and dominant (TT+TC vs. CC: OR = 1.469, 95% CI 1.148-1.880, p = 0.002), but not in Caucasian women.

*TNF-α rs1800629*

The summary for the association of TNF-α rs1800629 polymorphism with cervical cancer risk are shown in Table 3. Pooled data from all eligible studies indicated that the TNF-α rs1800629 polymorphism was associated with cervical cancer risk in overall population under four genetic models i.e., allele (A vs. G: OR = 1.277, 95% CI = 1.104-1.477, P = 0.001), homozygote (AA vs. GG: OR = 1.333, 95% CI = 1.062-1.674, P = 0.013), heterozygote (AG vs. GG: OR = 1.307, 95% CI = 1.064-1.605, P = 0.011), and dominant (AA+AG vs. GG: OR = 1.324, 95% CI = 1.104-1.587, P = 0.002). The subgroup analysis by ethnicity also showed that this polymorphism was associated with cervical cancer in Caucasian women under three genetic models i.e., allele (A vs. G, OR = 1.242, 95% CI = 1.043-1.478, P = 0.015); homozygote (AA vs. GG, OR = 1.586, 95% CI = 1.147-2.193, P = 0.005), and recessive (AA vs. AG+GG, OR = 1.569, 95% CI = 1.137-2.165, P = 0.006) and in African women under two genetic models i.e., heterozygote (AG vs. GG, OR = 1.670, 95% CI = 1.228-2.270, P = 0.001) and dominant (AA+AG vs. GG, OR = 1.453, 95% CI = 1.111-1.902, P = 0.006), but not in Asian women.

*MTHFR rs1801133*

The summary for the association of MTHFR rs1801133 polymorphism with cervical cancer risk are shown in Table 3. Pooled results showed that the MTHFR rs1801133 polymorphism was not associated with cervical cancer in overall population and by ethnicity.

*IL-12B rs3212227*

The summary for the association of IL-12B rs3212227 polymorphism with cervical cancer risk are shown in Table 4. Pooled data showed that the IL-12B rs3212227 polymorphism was not associated with cervical cancer globally. Stratified analysis by ethnicity revealed that this polymorphism was associated with cervical cancer in Asian women under two genetic models i.e., heterozygote (CA vs. AA: OR = 1.349, 95% CI 1.032-1.762, p=0.028) and dominant (CC+CA vs. AA: OR = 1.340, 95% CI 1.041-1.725, p =0.023).

*IL-6 rs1800795*

The summary for the association of IL-6 rs1800795 polymorphism with cervical cancer risk are shown in Table 4. Pooled data from all eligible studies showed that the IL-6 rs1800795 polymorphism was significantly associated with cervical cancer risk under four genetic models, i.e., allele (C vs. G: OR = 1.294, 95% CI 1.071-1.564, p= 0.007), homozygote (CC vs. GG: OR = 1.633, 95% CI 1.059-2.520, p= 0.027), dominant (CC+CG vs. GG: OR = 1.312, 95% CI 1.048-1.643, p= 0.018) and recessive (CC vs. CG+GG: OR = 1.592, 95% CI 1.268-1.999, p≤0.001). Moreover, subgroup analysis by ethnicity revealed an increased risk of cervical cancer in Asian women.

*TLR9 rs352140*

The summary for the association of TLR9 rs352140 polymorphism with cervical cancer risk are shown in Table 4. Pooled results showed that the TLR9 rs352140 polymorphism was not associated with cervical cancer in overall population and by ethnicity.

*Test of heterogeneity*

In the current meta-analysis the *χ*^2^ test and I^2^ statistics were used for assessing the heterogeneity of the included studies. Results indicate that there was statistical heterogeneity for XRCC3 rs861539, MTHFR rs1801133, IL-6 rs1800795, IL-12B rs3212227, TNF-α rs1800629, and TLR9 rs352140 polymorphisms under most genetic models. Thus, the random effect model was used for evaluating the pooled OR and 95% CI for those models. Subgroup analyses showed that ethnicity of participants might contribute to part of heterogeneity.

*Sensitivity Analysis*

The process of performing a meta-analysis involves a sequence of decisions and it is important to perform a sensitivity analysis in order to assess the impact of different decisions on pooled data. Thus, a sensitivity analysis was carried out by excluding a single study in turn on pooled ORs. The results showed that no individual study had an influence on the pooled OR all involved polymorphisms at XRCC3 rs861539, MTHFR rs1801133, IL-6 rs1800795, IL-12B rs3212227, TNF-α rs1800629, and TLR9 rs352140 polymorphisms, suggesting the stability of our findings. Moreover, excluding HWE deviated studies suggested that there were no independent studies that significantly influenced our pooled data.

*Publication Bias*

Begg’s funnel plot and Egger’s test were used for evaluating publication bias. As shown in Tables 3 and 4, the Egger’s test results showed that there was no publication bias for the XRCC3 rs861539, MTHFR rs1801133, IL-6 rs1800795, IL-12B rs3212227, TNF-α rs1800629, and TLR9 rs352140 polymorphisms under all five genetic models. Moreover, Begg’s funnel did not statistically revealed a significant publication bias in any of the models for all involved polymorphisms. Thus, the publication bias tests revealed that our pooled ORs were reliable.

**Table 1:** Characteristics of Studies Included in the Meta-Analysis

First Author/Year	Country	SOC	Genotyping	Case/Control	Cases					Controls						MAFs		HWE		
	(Ethnicity)		Technique		Genotypes			Allele		Genotypes			Allele							
XRCC3 rs861539					CC	CT	TT	C	T	CC	CT	TT	C	T						
He 2008	China(Asian)	PB	AS-PCR	200/200	177	19	4	373	27	182	17	1	381	19		0.047		0.391		
Xiao 2010	China(Asian)	PB	PCR-RFLP	158/164	82	59	17	223	93	115	41	8	271	57		0.173		0.097		
Settheetham-Ishida 2011	Thailand(Asian)	PB	PCR-RFLP	111/118	101	10	0	212	10	106	12	0	224	12		0.05		0.56		
Pérez 2013	Argentina(Caucasian)	PB	Sequencing	117/205	50	56	11	156	78	78	95	32	251	159		0.387		0.73		
Djansugurova 2013	Kazakhstan(Caucasian)	PB	AS-PCR	217/160	140	57	20	337	97	124	32	4	280	40		0.125		0.278		
Colacino-Silva 2017	Brazil(Mixed)	HB	PCR-RFLP	77/73	43	28	6	114	40	36	30	7	102	44		0.301		0.837		
Al-Harbi 2017	Saudi Arabia(Asian)	NA	PCR-RFLP	232/313	79	126	27	284	180	126	145	42	397	229		0.365		0.977		
MTHFR rs1801133					CC	CT	TT	C	T	CC	CT	TT	C	T						
Lambropoulos 2003	Greece(Caucasian)	HB	PCR-PFLP	21/91	11	8	2	30	12	42	37	12	121	61		0.335		0.403		
Sull 2004	Korea(Asian)	HB	SNapShot	246/454	73	115	58	261	231	153	221	80	527	381		0.42		0.989		
Kang 2005	Korea(Asian)	HB	PCR-PFLP	79/74	27	32	20	86	72	30	32	12	92	56		0.378		0.487		
Zoodsma 2005	Netherlands(Caucasian)	HB	TaqMan	636/592	357	230	49	944	328	273	262	57	808	376		0.318		0.608		
Ma 2006	China(Asian)	HB	PCR-PFLP	111/111	20	53	38	93	129	33	60	18	126	96		0.432		0.286		
Delgado 2006	Mexico(Mixed)	HB	PCR-PFLP	70/89	18	34	14	70	62	20	49	20	89	69		0.5		0.34		
Nandan 2008	India(Asian)	HB	PCR-PFLP	62/77	36	0	26	72	52	53	0	24	106	48		0.312		≤0.001		
Shekari 2008	India(Asian)	HB	PCR-PFLP	200/200	125	68	7	318	82	170	28	2	368	32		0.08		0.489		
Kohaar 2010	India(Asian)	HB	SNapShot	164/231	113	47	4	273	55	161	65	5	387	75		0.162		0.598		
Yang 2010	China(Asian)	HB	PCR-PFLP	391/382	229	85	77	530	234	182	166	34	536	234		0.306		0.658		
Mostowska 2011	Poland(Caucasian)	HB	PCR-PFLP	124/168	56	59	9	171	77	69	81	18	219	117		0.348		0.42		
Prasad 2011	India(Asian)	PB	PCR-PFLP	62/125	57	5	0	119	5	116	8	1	240	10		0.04		0.062		
Tong 2011	Korea(Asian)	HB	TaqMan	146/427	53	65	28	171	121	152	198	77	502	342		0.412		0.373		
Keyserling 2011	Germany(Caucasian)	HB	LDR-PCR	386/328	164	188	34	516	256	165	136	27	466	190		0.29		0.89		
IL-12B rs3212227					AA	AC	CC	A	C	AA	AC	CC	A	C						
Han 2008	Korea(Asian)	HB	SNaPShot	150/179	32	87	31	151	149	52	88	39	192	166		0.464		0.877		
Chen 2009	China(Asian)	PB	PCR-RFLP	404/404	127	199	78	453	355	150	185	69	485	323		0.4		0.357		
de Carvalho 2012	Brazil(Mixed)	PB	PCR-RFLP	162/76	100	49	13	249	75	31	37	8	99	53		0.349		0.531		
Roszak 2012	Poland(Caucasian)	PB	PCR-RFLP	405/450	212	174	19	598	212	289	151	10	729	171		0.19		0.055		
IL-6 rs1800795					GG	GC	CC	G	C	GG	GC	CC	G	C						
de Souza 2006	Brazil(Mixed)	PB	PCR-RFLP	56/253	24	32	0	80	32	148	102	3	398	108		0.213		0.001		
IL-6 rs1800795					GG	GC	CC	G	C	GG	GC	CC	G		C				
Gangwar 2009	India(Asian)	HB	ARMS-PCR	160/200	107	36	17	250	70	142	51	7	335		65		0.163		0.371
Grimm 2011	Austria(Caucasian)	HB	Sequencing	131/208	55	51	25	161	101	85	96	27	266		150		0.361		0.989
Junior 2012	Brazil(Mixed)	PB	Sequencing	115/115	72	39	4	183	47	67	37	11	171		59		0.257		0.093
Shi 2014	China(Asian)	HB	PCR-RFLP	518/518	160	253	105	573	463	181	259	78	621		415		0.401		0.348
Pu 2016	China(Asian)	HB	TaqMan	360/728	185	141	34	511	209	476	220	32	1172		284		0.195		0.309
Zidi 2017	Tunisia(African)	HB	qRT-PCR	112/164	81	25	6	187	37	133	25	6	291		37		0.113		0.002

**Table 2:** Characteristics of the Case-Control Studies Included in the Meta-Analyses

First Author	Country	SOC	Genotyping	Case/Control	Cases					Controls					MAFs	HWE
	Ethnicity		Method		Genotype			Allele		Genotype			Allele			
TNF-α rs1800629					GG	AG	AA	G	A	GG	AG	AA	G	A		
Jang 2001	Korea(Asian)	PB	PCR-RFLP	51/92	46	3	2	95	7	85	7	0	177	7	0.038	0.704
Calhoun 2002	USA(Caucasian)	HB	Sequencing	127/107	91	27	9	209	45	73	30	4	176	38	0.177	0.678
Stanczuk 2003	Zimbabwe(African)	PB	ARMS-PCR	103/101	74	28	1	176	30	81	18	2	180	22	0.108	0.41
Gostout 2003	USA(Caucasian)	HB	Sequencing	127/175	91	27	9	209	45	117	53	5	287	63	0.18	0.731
Duarte 2005	Portugal(Caucasian)	PB	PCR-RFLP	195/244	138	50	7	326	64	200	40	4	440	48	0.098	0.236
Deshpande 2005	USA(Caucasian)	HB	Sequencing	258/411	188	54	16	430	86	297	100	14	694	128	0.155	0.13
Govan 2006	South Africa(African)	HB	ARMS-PCR	244/228	174	62	8	410	78	172	46	10	390	66	0.144	0.005
Kohaar 2007	India(Asian)	HB	PCR-RFLP	120/165	94	22	4	210	30	150	15	0	315	15	0.045	0.54
Wang 2009	China(Asian)	PB	TaqMan	456/800	386	67	3	839	73	666	126	8	1458	142	0.088	0.457
Singh 2009	India(Asian)	HB	PCR-RFLP	150/162	122	17	11	261	39	147	11	4	305	19	0.058	≤0.001
Ivansson 2010	Sweden(Caucasian)	PB	TaqMan	1263/552	891	340	32	2122	404	396	138	18	930	174	0.157	0.169
Zu 2010	China(Asian)	HB	PCR	83/91	30	50	3	110	56	66	16	9	148	34	0.186	≤0.001
Wang 2011	China(Asian)	PB	PCR	186/200	149	30	7	328	44	144	46	10	334	66	0.165	0.019
Zuo 2011	China(Asian)	HB	PCR-RFLP	239/110	158	81	0	397	81	83	25	2	191	29	0.131	0.941
Wang 2012	China(Asian)	HB	PCR-RFLP	285/318	247	30	8	524	46	274	35	9	583	53	0.083	≤0.001
Barbisan 2012	Argentina(Caucasian)	HB	PCR-RFLP	122/176	87	32	3	206	38	126	46	4	298	54	0.153	0.483
Badano 2012	Argentina(Caucasian)	HB	Sequencing	56/113	44	10	2	98	14	101	12	0	214	12	0.053	0.551
Sousa 2014	Portugal(Caucasian)	PB	TaqMan	223/205	152	65	6	369	77	164	39	2	367	43	0.104	0.849
Zidi 2014	Tunisia(African)	PB	ARMS-PCR	130/260	55	33	43	143	117	141	35	84	317	203	0.39	≤0.001
Roszak 2015	Poland(Caucasian)	HB	HMR	362/399	217	123	22	557	167	263	125	11	651	147	0.184	0.397
TLR9 rs352140					GG	GA	AA	G	A	GG	GA	AA	G	A		
Pandey 2011	India(Asian)	PB	PCR-RFLP	200/200	59	115	26	233	167	59	112	29	230	170	0.425	0.039
Roszak 2012	Poland(Caucasian)	PB	PCR-RFLP	426/460	87	230	109	404	448	122	235	103	479	441	0.479	0.614
Lai 2013	China(Asian)	HB	PCR-RFLP	120/100	98	14	8	210	30	97	2	1	196	4	0.02	≤0.001
Bi 2014	China(Asian)	PB	PCR-RFLP	102/100	33	58	11	124	80	31	47	22	109	91	0.455	0.6
Zidi 2016	Tunisia(African)	PB	PCR-RFLP	130/260	42	48	40	132	128	83	117	60	283	237	0.456	0.134
Jin 2017	China(Asian)	HB	PCR-RFLP	420/842	208	160	52	576	264	543	257	42	1343	341	0.202	0.11
Xu 2017	China(Asian)	PB	TaqMan	345/330	135	163	47	433	257	131	152	47	414	246	0.373	0.785

**Table 3:** Results of the Association of XRCC3 RS861539, MTHFR rs1801133 and IL-12B rs3212227 Polymorphisms with Cervical Cancer Risk

Subgroup	Genetic Model	Type of Model	Heterogeneity		Odds Ratio				Publication Bias	
			I^2^ (%)	P_H_	OR	95% CI	Z_test_	P_OR_	P_Beggs_	P_Eggers_
XRCC3 rs861539										
Overall	T vs. C	Random	73.82	0.001	1.223	0.897-1.669	1.272	0.203	1	0.901
	TT vs. CC	Random	68.44	0.007	1.456	0.723-2.932	1.053	0.292	0.707	0.376
	TC vs. CC	Fixed	26.84	0.224	1.3	1.066-1.585	2.596	0.009	0.548	0.242
	TT+TC vs. CC	Random	58.55	0.025	1.27	0.935-1.726	1.53	0.126	0.763	0.452
	TT vs. TC+CC	Random	64.54	0.015	1.309	0.693-2.470	0.829	0.407	0.452	0.225
Asians	T vs. C	Fixed	60.39	0.056	1.302	1.076-1.576	2.716	0.007	1	0.862
	TT vs. CC	Fixed	58.94	0.088	1.457	0.918-2.314	1.595	0.111	1	0.446
	TC vs. CC	Fixed	14.62	0.319	1.441	1.113-1.867	2.768	0.006	0.308	0.474
	TT+TC vs. CC	Fixed	36.18	0.195	1.469	1.148-1.880	3.055	0.002	0.734	0.666
	TT vs. TC+CC	Fixed	61.31	0.075	1.165	0.754-1.801	0.689	0.491	1	0.375
Caucasians	T vs. C	Random	91.87	0	1.253	0.500-3.138	0.481	0.63	NA	NA
	TT vs. CC	Random	89.45	0.002	1.484	0.188-11.730	0.374	0.708	NA	NA
	TC vs. CC	Fixed	45.46	0.176	1.234	0.873-1.743	1.193	0.233	NA	NA
	TT+TC vs. CC	Random	83.94	0.013	1.248	0.551-2.826	0.532	0.595	NA	NA
	TT vs. TC+CC	Random	88.24	0.004	1.425	0.210-9.655	0.363	0.716	NA	NA
MTHFR rs1801133										
Overall	T vs. C	Random	73.78	≤0.001	1.132	0.956-1.341	1.434	0.151	0.62	0.232
	TT vs. CC	Random	49.57	0.013	1.212	0.924-1.590	1.388	0.165	0.964	0.802
	TC vs. CC	Random	77.11	≤0.001	0.985	0.755-1.284	-0.113	0.91	0.843	0.438
	TT+TC vs. CC	Random	79.6	≤0.001	1.095	0.842-1.423	0.677	0.498	0.752	0.215
	TT vs. TC+CC	Random	83.79	≤0.001	1.41	0.913-2.176	1.551	0.121	0.62	0.867
Ethnicity										
Caucasians	T vs. C	Fixed	45.38	0.16	1.071	0.891-1.287	0.733	0.464	1	0.431
	TT vs. CC	Fixed	8.4	0.336	0.996	0.637-1.559	-0.016	0.987	1	0.439
	TC vs. CC	Fixed	27.78	0.25	1.197	0.930-1.540	1.393	0.164	1	0.413
	TT+TC vs. CC	Fixed	44.89	0.163	1.162	0.912-1.480	1.214	0.225	1	0.406
	TT vs. TC+CC	Fixed	0	0.573	0.913	0.594-1.403	-0.415	0.678	1	0.47
Asians	T vs. C	Random	77.58	≤0.001	1.173	0.958-1.438	1.542	0.123	0.427	0.119
	TT vs. CC	Random	55.31	0.008	1.295	0.944-1.776	1.602	0.109	0.854	0.53
	TC vs. CC	Random	79.26	≤0.001	0.967	0.705-1.325	-0.211	0.833	0.945	0.264
	TT+TC vs. CC	Random	82.2	≤0.001	1.119	0.816-1.532	0.697	0.486	0.427	0.116
	TT vs. TC+CC	Random	86.09	≤0.001	1.594	0.961-2.642	1.806	0.071	0.582	0.924
IL-12B rs3212227										
Overall	C vs. A	Random	81.69	0.001	1.076	0.783-1.479	0.452	0.651	0.308	0.236
	CC vs. AA	Random	54.75	0.085	1.33	0.988-1.790	1.878	0.06	0.734	0.782
	CA vs. AA	Random	82.52	0.001	1.119	0.696-1.800	0.463	0.643	0.308	0.356
	CC+CA vs. AA	Random	83.68	≤0.001	1.125	0.704-1.799	0.492	0.623	0.308	0.317
	CC vs. CA+AA	Random	24.76	0.263	1.14	0.874-1.487	0.969	0.333	0.734	0.946
Asians	C vs. A	Fixed	0	0.87	1.166	0.988-1.377	1.812	0.07	NA	NA
	CC vs. AA	Fixed	0	0.932	1.323	0.941-1.860	1.61	0.107	NA	NA
	CA vs. AA	Fixed	0	0.454	1.349	1.032-1.762	2.191	0.028	NA	NA
	CC+CA vs. AA	Fixed	0	0.594	1.34	1.041-1.725	2.271	0.023	NA	NA
	CC vs. CA+AA	Fixed	0	0.507	1.086	0.807-1.461	0.542	0.588	NA	NA

NA, not applicable

**Table 4:** Summary Risk Estimates for Association of IL-6 rs1800795, TNF-α rs1800629 and TLR9 rs352140 Polymorphisms with Cervical Cancer Risk

Subgroup	Genetic Model	Type of Model	Heterogeneity		Odds Ratio				Publication Bias	
			I^2^ (%)	P_H_	OR	95% CI	Z_test_	P_OR_	P_Beggs_	P_Eggers_
IL-6 rs1800795										
Overall	C vs. G	Random	60	0.02	1.294	1.071-1.564	2.675	0.007	0.763	0.701
	CC vs. GG	Random	54.07	0.042	1.633	1.059-2.520	2.217	0.027	0.763	0.587
	CG vs. GG	Random	52.47	0.049	1.232	0.971-1.562	1.718	0.086	0.763	0.728
	CC+CG vs. GG	Random	53.01	0.047	1.312	1.048-1.643	2.371	0.018	1	0.583
	CC vs. CG+GG	Fixed	48.97	0.068	1.592	1.268-1.999	3.999	≤0.001	0.548	0.646
Asians	C vs. G	Fixed	65.97	0.053	1.395	1.230-1.582	5.195	≤0.001	1	0.793
	CC vs. GG	Fixed	56.74	0.099	1.951	1.472-2.584	4.656	≤0.001	1	0.375
	CG vs. GG	Fixed	66.54	0.05	1.289	1.077-1.543	2.769	0.006	1	0.613
	CC+CG vs. GG	Fixed	61	0.077	1.429	1.207-1.692	4.136	≤0.001	1	0.766
	CC vs. CG+GG	Fixed	53.87	0.114	1.736	1.339-2.251	4.166	≤0.001	0.296	0.205
TNF-α rs1800629										
Overall	A vs. G	Random	61.94	≤0.001	1.277	1.104-1.477	3.291	0.001	0.029	0.025
	AA vs. GG	Fixed	27.43	0.125	1.333	1.062-1.674	2.481	0.013	0.314	0.366
	AG vs. GG	Random	70.89	≤0.001	1.307	1.064-1.605	2.552	0.011	0.183	0.141
	AA+AG vs. GG	Random	67.34	≤0.001	1.324	1.104-1.587	3.03	0.002	0.097	0.056
	AA vs. AG+GG	Fixed	35.98	0.056	1.221	0.977-1.525	1.758	0.079	0.537	0.336
Asians	A vs. G	Random	78.48	≤0.001	1.403	0.970-2.029	1.798	0.072	0.035	0.062
	AA vs. GG	Fixed	43.54	0.088	1.089	0.670-1.770	0.343	0.731	1	0.54
	AG vs. GG	Random	82.21	≤0.001	1.469	0.895-2.411	1.521	0.128	0.173	0.267
	AA+AG vs. GG	Random	81.63	≤0.001	1.5	0.954-2.359	1.756	0.079	0.173	0.121
	AA vs. AG+GG	Random	50.71	0.048	1.04	0.487-2.217	0.1	0.92	0.901	0.647
Africans	A vs. G	Fixed	0	0.786	1.234	0.996-1.529	1.925	0.054	1	0.739
	AA vs. GG	Fixed	0	0.537	1.156	0.757-1.766	0.672	0.502	1	0.289
	AG vs. GG	Fixed	24.821	0.264	1.67	1.228-2.270	3.268	0.001	1	0.564
	AA+AG vs. GG	Fixed	0	0.585	1.453	1.111-1.902	2.725	0.006	1	0.766
	AA vs. AG+GG	Fixed	0	0.702	0.955	0.640-1.425	-0.225	0.822	1	0.185
Caucasians	A vs. G	Random	52.45	0.032	1.242	1.043-1.478	2.438	0.015	0.754	0.203
	AA vs. GG	Fixed	22.58	0.242	1.586	1.147-2.193	2.791	0.005	0.175	0.072
	AG vs. GG	Random	54.87	0.023	1.123	0.905-1.395	1.056	0.291	0.754	0.906
	AA+AG vs. GG	Random	52.8	0.031	1.201	0.982-1.469	1.787	0.074	0.916	0.501
	AA vs. AG+GG	Fixed	22.15	0.246	1.569	1.137-2.165	2.744	0.006	0.348	0.079
TLR9 rs352140										
Overall	A vs. G	Random	84.19	≤0.001	1.231	0.946-1.603	1.545	0.122	0.763	0.892
	AA vs. GG	Random	77.72	≤0.001	1.341	0.834-2.154	1.211	0.226	0.548	0.773
	AG vs. GG	Random	57.65	0.028	1.236	0.962-1.588	1.658	0.097	1	0.925
	AA+AG vs. GG	Random	72.99	0.001	1.299	0.967-1.745	1.74	0.082	1	0.939
	AA vs. AG+GG	Random	76.74	≤0.001	1.226	0.810-1.856	0.962	0.336	0.763	0.965

## Discussion

Genetic factors have been shown to influence the susceptibility of patients to various diseases and have attracted increasing attention (Motamedi et al., 2012; Mazaheri et al., 2014; Kabiri Rad et al., 2018). Several efforts have been made to identify the genetic susceptibility factors underlying development of cervical cancer. However, only a few polymorphisms have shown consistency among studies. In this meta-analysis, the association of XRCC3 rs861539, MTHFR rs1801133, IL-6 rs1800795, IL-12B rs3212227, TNF-α rs1800629 and TLR9 rs352140 polymorphisms with susceptibility to cervical carcinoma was assessed by including all relevant studies.

Yuan et al., (2021) in a meta-analysis based on 15 studies with 5,740 cases and 9,931 controls revealed that there was no significant association between the XRCC3 Thr241Met and the risk of gynecological malignancies. However, their subgroup analysis by ethnicity showed that XRCC3 Thr241Met was associated with an increased risk of gynecological malignancies in Asians. Moreover, their stratified analysis by cancer type indicated that XRCC3 Thr241Met associated with cervical cancer in Asians (CT vs. CC: OR=1.50, 95%CI=1.04-2.14; TT vs. CC: OR=3.14, 95%CI=1.38-7.14; CT+TT vs. CC: OR=1.64, 95% CI=1.17-2.31). Abbas et al., (2010) in a case-control study with 260 cervical cancer cases and 265 controls evaluated the association of XRCC1+399A/G, XRCC2+31467G/A and XRCC3+18067C/T polymorphisms with cervical cancer in Indian women. Their results showed that XRCC2+31479G/A and XRCC3+18067C/T polymorphisms were not associated with cervical cancer. However, they showed that he XRCC1+399A/G is linked with cervical cancer in the Indian population. Al-Harbi et al., (2017) in a study among 232 cervical cancer cases and 313 control subjects evaluated the association of CDKN1A C31A, ATM G1853A, HDM2 T309G, TGFB1 T10C, XRCC1 G399A, and XRCC3 C241T with cervical cancer among Saudi Arabian women. They showed that the TGFB1 T10C and XRCC1 G399A polymorphisms were associated with cervical cancer risk.

Our pooled results showed that the MTHFR rs1801133 polymorphism was not associated with cervical cancer in overall population and by ethnicity. Silva et al., (2019)reported that there were no differences in the genotypic and allelic distribution of MTHFR C677T polymorphism between remission (with the presence of pre-neoplastic lesions) and Persistence (with the presence of pre-neoplastic lesions). The same authors, in another study showed that MTHFR C677T polymorphism was not associated with cervical cancer and HPV infection (Silva et al., 2019). However, Sohrabi et al., in a case-control study evaluated the association of MTHFR A1298C and C677T variants among in 50 cervical intraepithelial neoplasia cases, 98 HPV-positive subjects and 47 non-cancerous/non-HPV patients as healthy controls. Their results showed that MTHFR 1298 CC is more likely to be a potential risk factor for HPV-cervical cancer progression (Sohrabi et al., 2020). Zhou et al., (2020) indicated that the MTHFR rs4846048 enhanced the risk of cervical cancer through association with miR-522. Gong et al., (2018) reported that MTHFR C677T polymorphism was not associated with the risk of cervical cancer or cervical intra-epithelial neoplasia, while, the MTHFR A1298C polymorphism could increase the risk of both cervical cancer and cervical intra-epithelial neoplasia.

In the current study pooled data indicated that the TNF-α rs1800629 polymorphism was associated with cervical cancer risk. Wang et al., (2012) in meta-analysis based 27 studies showed that both TNF-α -238 and -308 G/A polymorphisms could be used to identity individual with elevated susceptibility to cervical cancer in by ethnicity (Bi, 2014). Behboodi et al., (2021) in a study among 153 Iranian cervical cancer cases and 292 free cancer subjects demonstrated that TNF-α rs1800629 was associated with increased level and risk of developing cervical cancer. However, Duvlis et al., (2020) reported that TNF-a-238G/A and TNF-a-308 G/T polymorphisms were not associated with the risk of HPV associated cervical intraepithelial lesions or cervical cancer cases in Macedonian women compared to controls. Moreover, Traore et al., (2020) revealed that TNF-308 G/A or IL18-607C/A polymorphisms were not associated with HPV infection among Burkina Faso women.

Pooled data from all eligible studies showed that the IL-6 rs1800795 polymorphism was significantly associated with cervical cancer risk in overall population and among Asian women. Similarly, Duan et al., (2018) in a meta-analysis based on 7 studies showed that the IL-6 rs1800795 polymorphism is associated with risk of cervical cancer in overall. Pu et al., (2016) in a study with 360 cervical cancer cases and 728 healthy subjects showed that this polymorphism is risk factor for cervical cancer development in Chinese women. de Souza et al., (2006) in a case-control study with 56 cases and 253 controls evaluated the association of IL-6 rs1800795 polymorphism with cervical cancer in a Brazilian population. In 2017, Zidi et al., (2017) in a case-control study evaluated the effects of six different genetic variants atIL-6 with risk of cervical cancer. The study revealed that the IL-6 rs1800795 polymorphism has a has a protective role in cervical cancer development in Tunisian women.de Lima Júnior et al., (2016) revealed that the IL-6 rs1800795 polymorphism was not associated with HPV infection and healthy controls in in Brazilian women. The current meta-analysis data showed that the IL-12B rs3212227 genetic variant was not associated with susceptibility to cervical cancer, while; stratified analysis showed that this polymorphism was associated with cervical cancer risk among Asian women. Zheng et al., (2017) in a meta-analysis based on 33 articles with 10,587 cancer cases and 12,040 healthy subjects assessed the genetic association of IL-12B rs3212227 with cancer risk. Their pooled data indicated that IL-12B rs3212227 polymorphism was associated with cancer risk in overall. However, subgroup analysis by cancer type showed that IL-12B rs3212227 was not associated with cervical cancer. In 2016, Chang et al., (2015) in a meta-analysis based on 5 studies with 2552 cervical cases and 2232 healthy subjects showed that this polymorphism did not associate with risk of cervical cancer. In 2012, de Carvalho et al., found that the IL-12B rs3212227 variant has a protective role in development of cervical cancer in Brazilian women (Do Carmo Vasconcelos De Carvalho et al., 2012). Similarly, Han et al., (2008) found that IL-12B rs3212227 did not associate with cervical cancer risk in Korean population. However, Roszak et al., (2012) showed that this polymorphism was associated with increased risk of cervical cancer among polish women.

The current meta-analysis data showed that TLR9 rs352140 was not associated with susceptibility to cervical cancer. Nath et al., (2020) in a study showed that TLR4/9 polymorphisms are associated with increased HPV16/18 infection susceptibility and cervical squamous cell carcinoma risk among the women of Jharkhand state. In another study in India showed the TLR4 and TLR9 polymorphisms and haplotypes with hrHPV infection and cervical cancer risk (Pandey et al., 2019). Martínez-Campos et al., (2017) in a case-control study revealed that TLR9 rs187084 is a risk factor for HPV infection, squamous intraepithelial cervical lesion, and uterine cervical neoplasm in Mexican female population. Jin et al., (2017) also evaluated the association of some variants at Toll-like receptors gene with cervical cancer among 420 Chinese cervical cancer patients and 842 controls. Their results showed that mutant alleles of TLR2 rs3775290, TLR4 rs7873784, and TLR9 rs352140 were associated with increased cervical cancer risk. Similarly, Yang et al., (2020) in meta-analysis based on eleven studies indicated that the TLR9 rs187084 and rs352140 polymorphisms may contribute to development of cervical cancer, but not TLR2-196 to -174 del/ins polymorphism.

In summary, our pooled data indicated that the XRCC3 RS861539, TNF-α rs1800629, and IL-6 rs1800795 genetic variants were associated with susceptibility to cervical cancer globally. However, the MTHFR rs1801133, IL-12B rs3212227 and TLR9 rs352140 variants were not associated. Larger and more rigorous studies among different ethnicities are needed to further evaluate these associations with cervical cancer.

## Author Contribution Statement

Seyedeh Fatemeh Parsaeian, Fatemeh Asadian, Mojgan Karimi-Zarchi: conceptualization, investigation. Seyed Alireza Dastgheib, Sepideh Setayesh: Software, original draft preparation. Atiyeh Javaheri, Razieh Sadat Tabatabaie: Investigation. Fatemeh Asadian: Investigation, writing. Fatemeh Asadian, Hossein Neamatzadeh: Methodology, software. Hossein Golestanpour, Hossein Neamatzadeh: Formal analysis, investigation. Hossein Golestanpour: Project administration. Atiyeh Javaheri, Razieh Sadat Tabatabaie: Writing, reviewing, editing.

## Ethics approval

This article does not contain any studies with human participants or animals performed by any of the authors.

## Consent to participate

Not applicable for this manuscript.

## Availability of data and material

The datasets generated during and/or analyzed during this study are available from the corresponding author on reasonable request.

## Conflicts of interest

The authors declare that they have no conflict of interest.
